# The Hydrophobic Temperature Dependence of Amino Acids Directly Calculated from Protein Structures

**DOI:** 10.1371/journal.pcbi.1004277

**Published:** 2015-05-22

**Authors:** Erik van Dijk, Arlo Hoogeveen, Sanne Abeln

**Affiliations:** Computer Science Department, Centre for Integrative Bioinformatics (IBIVU), VU University, Amsterdam, Netherlands; Max Planck Institute for Biophysical Chemistry, GERMANY

## Abstract

The hydrophobic effect is the main driving force in protein folding. One can estimate the relative strength of this hydrophobic effect for each amino acid by mining a large set of experimentally determined protein structures. However, the hydrophobic force is known to be strongly temperature dependent. This temperature dependence is thought to explain the denaturation of proteins at low temperatures. Here we investigate if it is possible to extract this temperature dependence directly from a large set of protein structures determined at different temperatures. Using NMR structures filtered for sequence identity, we were able to extract hydrophobicity propensities for all amino acids at five different temperature ranges (spanning 265-340 K). These propensities show that the hydrophobicity becomes weaker at lower temperatures, in line with current theory. Alternatively, one can conclude that the temperature dependence of the hydrophobic effect has a measurable influence on protein structures. Moreover, this work provides a method for probing the individual temperature dependence of the different amino acid types, which is difficult to obtain by direct experiment.

## Author Summary

In general, proteins become functional once they fold into a specific globular structure. On folding, hydrophobic amino acids get buried inside the protein such that they are shielded from the water; this hydrophobic effect makes a protein fold stable. However, the strength of the hydrophobicity is known to be strongly temperature dependent, leading for example to lower stability at lower temperatures (cold denaturation). Nevertheless, it is difficult to quantify the temperature dependence for hydrophobic amino acids. Here we are able to estimate the strength of the hydrophobic effect, by analysing the positions of a large number of amino acids from protein structures experimentally determined at different temperatures. For each amino acid type, we use the ratio between the number of residues at the inside and at the surface of the folded structures as a measure for its hydrophobicity. This approach shows that the hydrophobic effect becomes weaker at lower temperatures, as expected from theoretical predictions. Understanding the temperature dependence for amino acids, can help to make proteins (or enzymes) stable at a specific temperature range. For example, the design of enzymes that are stable and functional at low temperatures may benefit from this work.

## Introduction

When a protein folds, hydrophobic amino acids get buried inside the protein to form a hydrophobic core. Inside this core the hydrophobic side chains are shielded from the water. The tendency of hydrophobic groups to cluster together when they are put into water—or the hydrophobic effect—is the most important driving force in protein folding. Note that there are several factors that contribute to the overall stability of a folded protein: for example the formation of hydrogen bonds between backbone atoms (secondary structure) and side chains; the formation of salt bridges between charged amino acids and the burial of hydrophobic side chains upon folding. It is thought that this hydrophobic force gives the single largest contribution to the stability of most protein folds [1]. Moreover, the positioning of hydrophobic clusters in the sequence may affect the folding pathway and dynamics e.g. [2, 3]. Note that these stabilizing forces are partially compensated by the decrease in chain entropy upon folding.

**Fig 1:**
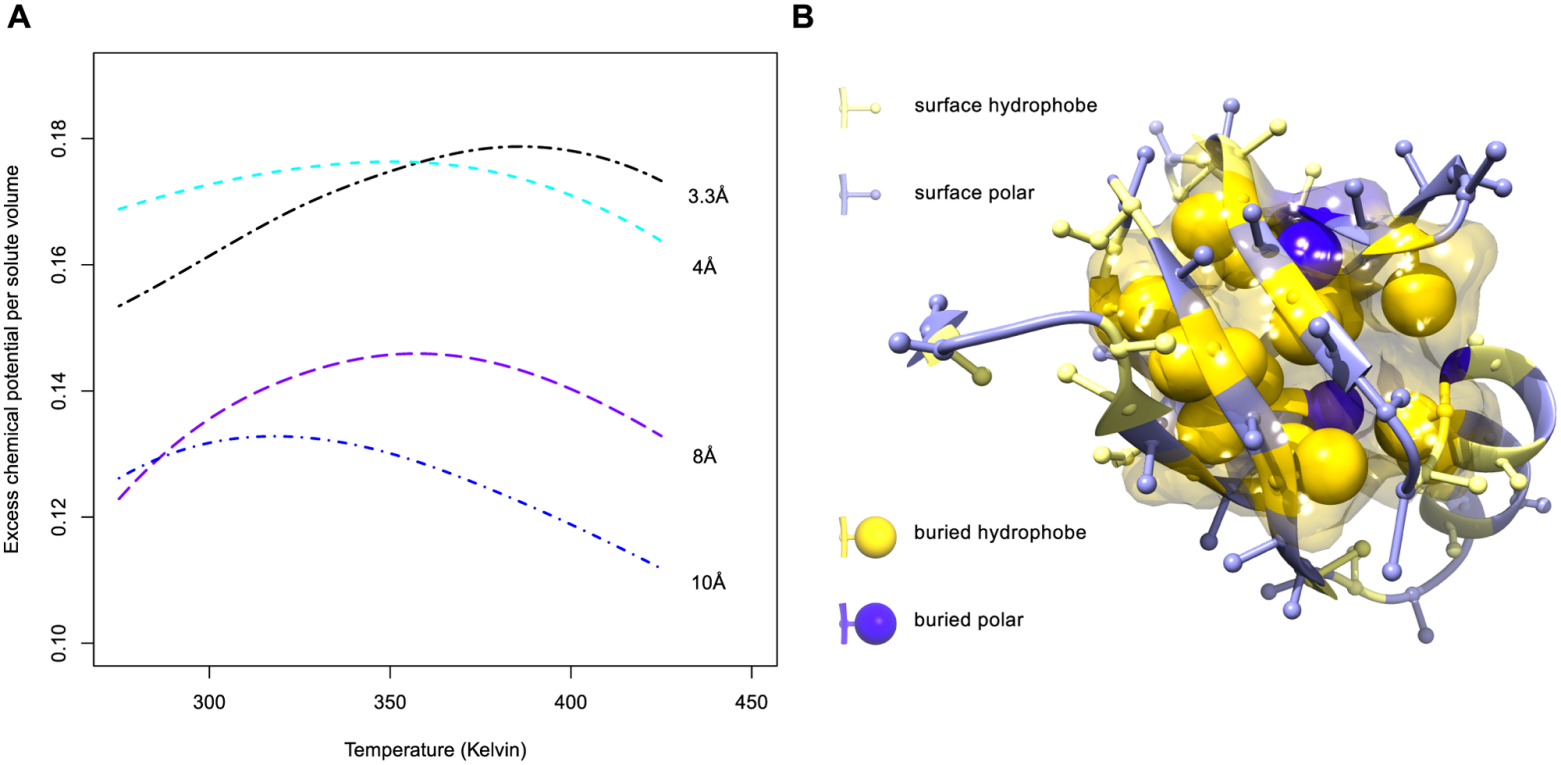
Length scale dependence of hydrophobic effect from calculations by Huang and Chandler [10] (A). The cost of making a cavity in the water with a radius of the given size against temperature is plotted. The position of the maximum depends on the size (radius) of the solute. Small solutes with a radius of 4 Å have a peak at around 70°C, whereas larger particles with a radius of 10 Å have a peak around 40°C. **An example protein structure: PDB-ID: 2K5I (B).** We estimate free energies of transfer from the hydrophobic core to the surface of the protein by comparing the number of hydrophobic amino acids on the surface (small yellow spheres), to the number of buried hydrophobics (large yellow spheres), to the number of polar amino acids on the surface (small blue spheres) and to the number of buried polar amino acids (large blue spheres).

Hydrophobicity is a result of the collective behaviour of the water molecules and ‘oily’ groups. In essence the water-hydrophobe interface is unfavourable compared to water-water or hydrophobic-hydrophobic interactions. The free energy difference upon burial of hydrophobic groups is partially entropic and partially enthalpic, causing a distinct temperature dependence [4, 5]. Even though the exact molecular cause for these enthalpic and entropic contributions is the focus of active research [6, 7] and can change depending on the type of protein [7], the resultant temperature dependence can be measured experimentally for several different non-polar substances [8, 9]. From such measurements, models and theory we know that the hydrophobic force peaks between 30–80°C and becomes weaker at both lower and higher temperatures, see Fig 1A.

Since hydrophobicity is such a large contributor to protein stability, the temperature dependence of the hydrophobic effect has important consequences. Firstly, some proteins do not only unfold at high temperatures, as can be explained through the entropy of the chain, but also at low temperatures (cold denaturation) [11]. This effect is thought to be a consequence of hydrophobicity becoming weaker at low temperatures [12]. Secondly, alternate states of intrinsically disordered proteins may become more favourable at different temperatures due to this effect [13]. Thirdly, protein-protein and protein-substrate interactions—if dominated by hydrophobic interactions—may also be sensitive to temperature changes.

It is essential to quantify the temperature dependence if one wants to model and predict the stability of folded proteins and protein interactions over a large range of temperatures. For industrial purposes, proteins or enzymes that can be active over a wide temperature range are of crucial importance. To achieve this, proteins from species that live at extreme temperatures, thermophiles and psychrophiles, have been used and adapted extensively for biocatalysis [14, 15]. Understanding and quantifying the hydrophobic temperature dependence for specific amino acids is essential if one wants to predict thermostability of proteins.

Earlier, Folch *et al*. [16] showed that temperature dependent pairwise potentials for amino acids can help to predict the melting temperature of homologous pairs of proteins. More recently, this study was extended to also predict stability at low temperatures [17]. In this work we focus on the temperature dependence of the effective interactions between hydrophobic amino acids and water.

Even though this temperature dependence has important consequences, it is often not considered due to practical concerns. The temperature dependence is typically not included in interaction potentials for protein structure prediction or coarse grained simulations; such potentials do not model the water molecules explicitly or in enough detail to capture this effect. It is difficult to measure the temperature dependence for specific amino acids by experiments, under physically relevant conditions. In other words, it is difficult to measure the difference in free energy between the folded and unfolded chain for separate amino acids. In this work we show that it is possible to obtain this temperature dependence for specific amino acids by mining a large set of protein structures resolved by Nuclear Magnetic Resonance (NMR).

Physically or chemically relevant quantities can be obtained by averaging over a large set of structures. For example, specific bond lengths, the most favourable dihedral angles or approximate hydrophobicities for different amino acid types can be obtained by taking an ensemble average over a set of protein structures. More specifically, hydrophobicity scales for the different amino acid types may be obtained using physicochemical properties [18], or by calculating how often we find each residue type exposed to the solvent at the surface of a protein [18–21]. Different approaches give slightly different results—and a somewhat different ranking between the residues—but do agree overall. Hydrophobicity scales are useful for a wide range of problems involving structure prediction: from predicting the severity of a mutation to disorder prediction and full structure prediction e.g. [22–27].

Estimates for pairwise free energies between amino acid types have been obtained by mining protein structures. A pairwise interaction potential may be calculated by counting the number of contacts made between different types of amino acids [16, 28, 29]. More recently, this method has been further developed to allow the extraction of interactions between the solvent and the different types of residues, as well as the pairwise interactions [30]. Knowledge-based amino acid pair-potentials are used in structure prediction [31], coarse-grained protein simulations [32–35] and protein-protein docking methods [36]. Recently, a knowledge based amino acid pair potential with a temperature dependence has also been used to predict the thermostability of proteins [17].

In this work, we estimate the hydrophobic effect as the free energy cost for transferring a hydrophobic amino acid from the core of the protein to the water exposed surface, see Fig 1B. We use three distinct approaches to estimate these transfer free energies. Firstly, we use a previously validated approach to derive a statistical pair potential between amino acids to extract free energy estimates for the hydrophobic interaction. This *contact* based method has been shown to yield hydrophobicity estimates that give physically realistic results upon simulation. Secondly, we use a more direct approach that calculates propensities for *surface* accessibility for each of the amino acids; this method is similar to other approaches that derive knowledge based hydrophobicity scales [18–20]. Thirdly, we use an *area* based approach that considers the amount of exposed surface area per amino acid. The three approaches give similar results, and show significant temperature dependence for hydrophobic amino acids in line with expectations from theory and measurements on small hydrophobic particles.

## Results/Discussion

**Fig 2:**
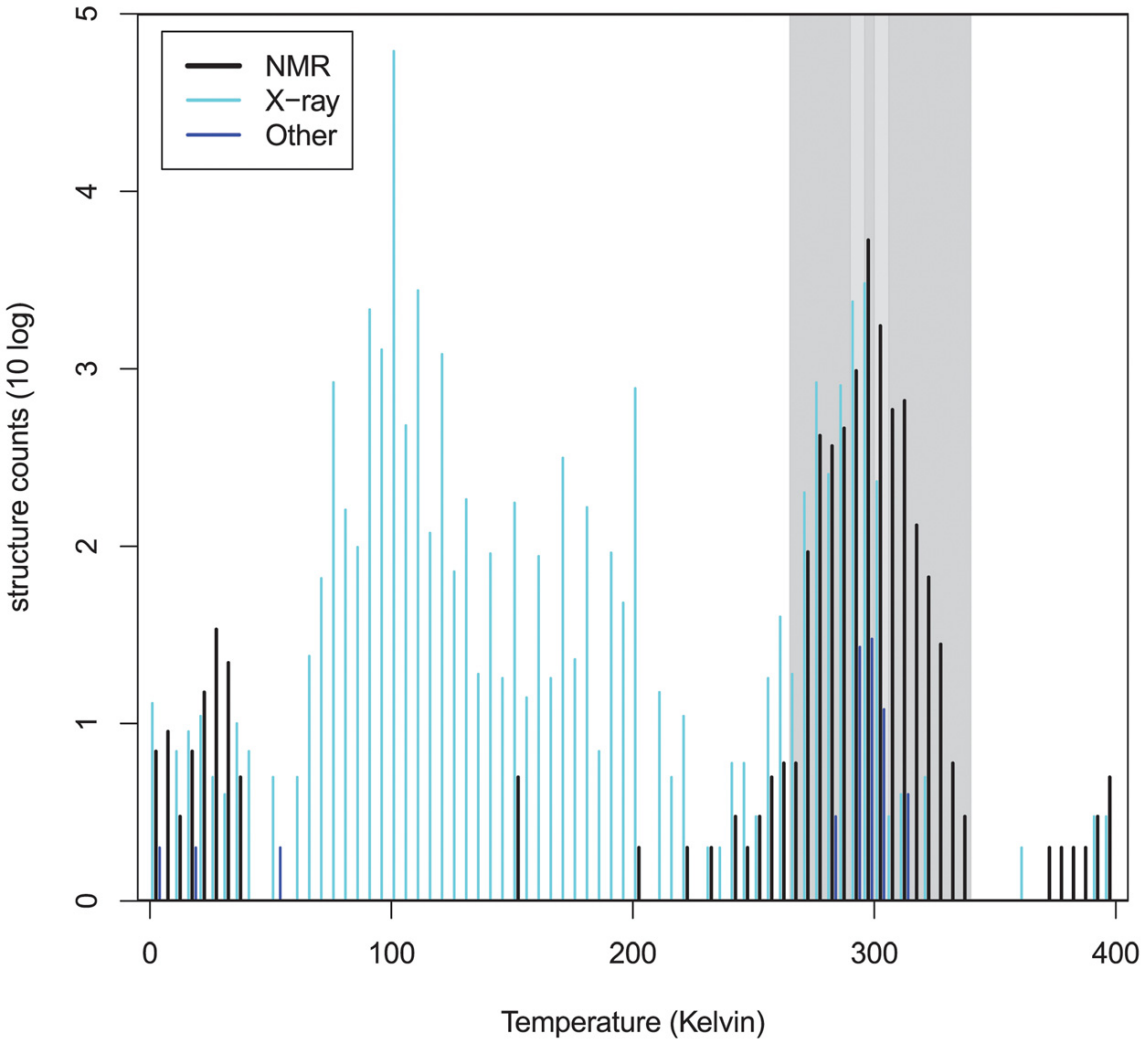
Distribution of temperatures at which experimental protein structures were resolved. All acquisition temperatures of structures as of April 2014 available in the PDB are shown. The 80,662 X-ray diffraction structures are centred around 100 K, while the 10,969 NMR structures show a peak at room temperature (300 K). Note that the small peak of NMR data just above absolute zero may be temperatures entered in celsius instead of kelvin; this data is not used in this study. Temperature bins, as given in Table 1, are indicated in different shades of grey.

In order to extract the hydrophobic temperature dependence from experimentally determined protein structures, it is important to choose the set of structures carefully. Firstly, we explored the contents of the Protein DataBank (PDB), [37], containing over 96k structures. Fig 2 shows the temperature distribution of available protein structures determined by X-ray crystallography and nuclear magnetic resonance (NMR). For this study we only use structures determined by NMR, as these experiments can be performed on soluble proteins at the temperature range of interest for the hydrophobic effect. This makes it possible to probe temperature dependent effects in proteins; for example a temperature induced transition [38] and cold denaturation [11] have been observed using this technique.

In order to obtain estimates for the solvation free energies of different types of amino acids at different temperatures, we divided the data into five temperature bins, see Fig 2 and Table 1. The bins were chosen symmetrically around the peak at room temperature (300 K), to balance the number of structures in each bin.

**Table 1:** Selected protein structures.

temperature range	chains in PDB	chains select-25	chains after filters
265–290	1421	259	207
291–296	1440	378	344
297–299	4689	1095	1033
300–305	1864	618	560
306–340	1361	470	412

The number of NMR chains as present in the PDB before and after filtering is shown for different temperature bins (in kelvin). At the first stage of filtering, sequence bias was removed using PDB-select-25. At the last filtering step, chains were removed when they were not compatible with DSSP or when they had multiple and different acquisition temperatures.

We set out to explore if we can observe the temperature dependence of the hydrophobic effect by analysing this filtered set of protein structures. Protein structures determined by NMR at different temperatures were used to obtain free energy estimates for the transfer of amino acids from the core of the protein to the surface. Under the assumption of random mixing, the transfer free energy estimates can be estimated through statistical methods [16, 28–30]. We investigate three methods, 1) a *contact* based calculation which has been shown to give a reasonable attraction [30], 2) a direct calculation of propensities to *surface* exposure 3) an *area* based calculation that incorporates the accessible surface area in a continuous measure of hydrophobicity, see Methods for details.

**Fig 3:**
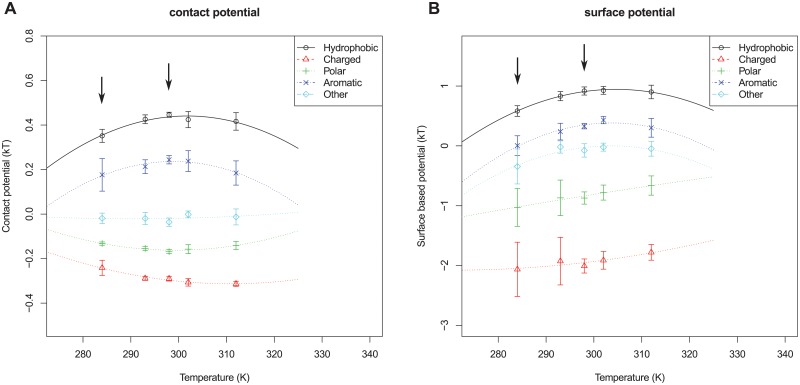
Raw free energies of transfer for classes of amino acids. Contact based (A) and surface based (B) free energies are shown for different classes of amino acids. Points show the free energy estimates for each temperature bin, lines are fitted with a parabola, consistent with the potentials found in [10]. Arrows indicate the bins used to test the significance of the temperature dependence.

Firstly, we investigate whether the raw free energy estimates are dependent on the temperature. To further increase the statistical accuracy, amino acids are divided into five classes: hydrophobic, charged, polar, aromatic and other, see Table 2. Fig 3 shows a surprisingly clear temperature dependence for the different hydrophobic amino acids: at lower temperatures the hydrophobic effect becomes weaker. This is in line with expectations from experiments and theory [4, 5]. The results for the area based potential are very similar to the results of the contact based potential (see S7–S14 Figs).

**Table 2:** Amino acid class definition.

Class	Amino Acids
Hydrophobics	ALA, ILE, LEU, MET, VAL
Aromatics	HIS, PHE, TRP, TYR
Charged	ARG, ASP, GLU, LYS
Polar	ASN, GLN, SER, THR
Other	CYS, GLY, PRO

To test if this temperature dependence is indeed significant, we resampled the protein structures using random temperature labels. From this procedure p-values were calculated to determine the significance of the free energy difference. Table 3 shows the difference in transfer energy (ΔΔ*G*) and p-values between the lowest temperature bin (265–290K) and room temperature (297–299K). Clearly, the temperature trend for the hydrophobic residues is significantly stronger than one would expect from random fluctuations. The standard error to the mean is estimated from the deviations in the potential obtained as indicated in the results by splitting the data set into five parts and recalculating the potentials for each part.

**Table 3:** Significance of hydrophobic temperature dependence pooled.

amino acid class	p-value contacts	p-value surface	ΔΔ*G* contacts	ΔΔ*G* surface
hydrophobic	< 0.01	< 0.01	0.10	0.32
polar	< 0.01	0.23	-0.05	0.13
charged	< 0.01	0.80	-0.06	-0.04
aromatic	0.04	< 0.01	0.06	0.32
other	0.32	< 0.01	0.02	0.41

The difference in free energy estimates (ΔΔ*G*) between the lowest temperature bin (265–290K) and room temperature (297–299K) is shown together with its significance (p-value) for each class of amino acids. The significance was tested using a resampling procedure. The amino acids are pooled according to defined classes; the free energy estimates are not reference corrected.

Fig 3 also shows that the surface based potentials give larger absolute differences in free energies than the contact based potentials. This can most likely be explained by the strict cutoff (7% accessible surface area) in the surface based potential compared to the more gradual calculation of the contact based potential; charged and polar amino acids are rarely entirely buried and give therefore a very strong signal for the surface based measure. The relative hydrophobicity, however, is consistent between the three methods, showing our results are qualitatively independent of the method of derivation for the potential.

The results in Fig 3 show a slight temperature dependence for charged (and polar) amino acids. For the surface based potential, however, this effect is not significant (Table 3).

Our transfer free energy estimates are calculated under the assumption of a random mixing model; this provides us with *relative* transfer free energies for each type of amino acids. This means it is not trivial to compare the free energy differences between different temperature bins. The temperature dependence of the hydrophobic residues could cause the shift of the polar and charged amino acids. In order to enable comparison at different temperatures, we set a reference state for the free energy estimates. The reference state is an important part of the potential, and can determine the accuracy of a potential in structure validation [39].

As we are here particularly interested to compare the transfer free energies between different temperatures it is desirable that our reference does not have any temperature dependent interaction with the solvent. Betancourt and Thirumalai [29] and Buchete *et al*. [27] use Threonine, a small water-like polar amino acid, as a reference in the calculation for their amino acid pair-potential. In our case, as the number of structures available is limited, choosing a single amino acid as reference will propagate noise through the results. Instead, we pool all the charged and hydrophilic amino acids for each temperature bin, and use those as a reference potential (see Table 2). Even though it is known that polar and charged residues can have a temperature dependent interaction with the solvent and that this interaction can have consequences for protein structure and stability (see for example Refs. [40, 41]), comparing *raw* estimates (Fig 3) with *reference corrected* estimates (S1 and S4 Figs) shows that this correction does not change the relative trends, see Methods for further details.

**Fig 4:**
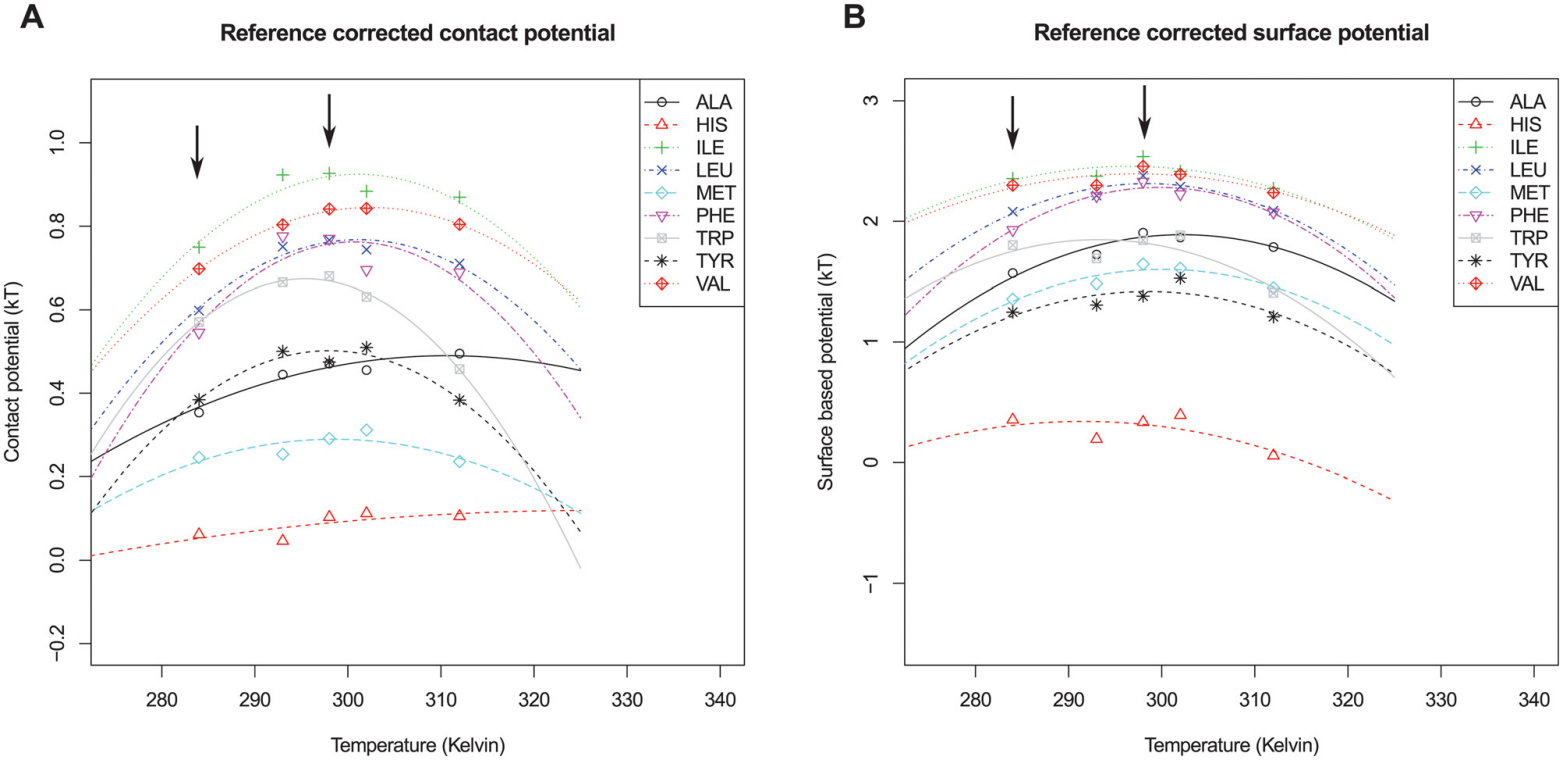
Reference corrected free energies of transfer for hydrophobic amino acids. Contact based (A) and surface based (B) free energies are shown for hydrophobic and aromatic amino acids. The free energies are corrected by setting a reference of the polar and charged amino acids. Points show the free energy estimates for each temperature bin and lines are fitted with a parabola. Arrows indicate the bins used to test the significance of the temperature dependence.

Fig 4 shows estimates for the corrected transfer free energies for all hydrophobic and aromatic amino acids individually, with the polar and charged amino acids as a reference. Results for all amino acids, with and without reference correction are shown in S2, S3, S5 and S6 Figs. The hydrophobicity becomes weaker at lower temperatures, showing the results from the ‘raw’ estimates hold up. Again, the significance of the temperature dependence of each hydrophobic amino acid type is examined. For almost all hydrophobic amino acids the free energy estimates have a significant temperature dependence (Table 4). Note that the correction to a reference of polar and charged amino acids was also performed in the resampling procedure to obtain statistical significance

**Table 4:** Significance of hydrophobic temperature dependence.

amino acid (class)	p-value contacts	p-value surface	ΔΔ*G* contacts	ΔΔ*G* surface
ALA	0.03	< 0.01	0.12	0.38
CYS	< 0.01	< 0.01	0.32	0.67
GLY	0.42	0.12	0.03	0.11
ILE	< 0.01	< 0.01	0.20	0.19
LEU	< 0.01	< 0.01	0.19	0.32
MET	0.68	0.09	0.03	0.19
PHE	< 0.01	< 0.01	0.23	0.36
PRO	0.62	< 0.01	0.07	0.46
TRP	0.23	0.21	0.15	0.16
TYR	0.34	0.08	0.10	0.19
VAL	0.02	< 0.01	0.15	0.16

The difference in free energy estimates (ΔΔ*G*) between the lowest temperature bin (265–290K) and room temperature (297–299K) is shown together with its significance (p-value) for each class of amino acids. The significance was tested using a resampling procedure. The hydrophobic and aromatic amino acids are shown and are reference corrected with respect to the charged and polar amino acids.

Fig 4 also shows that the estimated transfer free energies show a very similar trend with respect to temperature to those that have been measured for hydrophobic particles [4] or obtained by calculation according to LCW-theory [10, 42]. For clarity, we fitted parabolas through the estimated transfer free energies, which is a reasonable approximation for trends calculated from theory and observed in experiment (see S15 Fig). It can be observed that the free energies for the hydrophobic amino acids show a maximum of around 310–350 kelvin for both the surface and contact based free energy estimates; this is slightly lower than what is expected from theory (see for comparison Fig 1A)

Due to the lack of data at higher temperatures (*T* > 320*K*), it is difficult to estimate a precise maximum for the transfer free energies. Nevertheless, an interesting trend may be observed from Fig 4. Larger amino acids, for example Tryptophan, have a maximum at lower temperatures compared to smaller amino acids such as Alanine. Again, this trend is consistent with theory and experiments [10], where the transfer free energy of larger particles shows a maximum at lower temperatures.

Overall, we can conclude that the temperature dependence of the hydrophobic effect has a measurable influence on protein structures determined by NMR. The effect we find appears to be on the right order of magnitude in comparison with theory for the hydrophobic effect and known cold denaturating behaviour of proteins (see S2 Text). The results show that structures determined at lower temperature have more exposed hydrophobic surface area. This suggests that at these temperatures the structures already become more open, as has been observed for some specific proteins (e.g. [43]). It would be very interesting to investigate if these low temperature structures are more flexible and dynamic than the same structures obtained at room temperature.

### Conclusion

In this work we set out to investigate whether the hydrophobic temperature dependence could be obtained by mining a large set of protein structures resolved by NMR. We used a contact based, an area based and a surface based approach to obtain free energy estimates for the transfer of an amino acid out of the hydrophobic protein core onto the water exposed surface. We find a surprisingly clear trend for the free energy estimates with respect to the temperature: the hydrophobic effect becomes weaker at lower temperatures, as is expected based on theory, simulations and experiments. Alternatively, one can conclude that the temperature dependence of the hydrophobic effect has indeed a measurable influence on protein structures. Despite the sparseness of the data, and the inconsistencies in reporting of experimental temperatures, we find that the observed trend holds and is significant regardless of the precise method used to estimate the transfer free energies, the specific groupings of amino acids or the chosen reference.

## Methods

### Data collection

The temperature (in kelvin) at which the experiment is performed can be found in the mandatory ‘acquisition data’ section of PDB files. Several filters were applied. Some structures were filtered out because no temperature was entered or because they were given several temperatures from multiple data collection sessions. In order to get representative statistics for amino acid composition, it is important to remove any bias in the PDB for large sequence families. To take out this redundancy we used PDB filter-select 25% [44–46]. Table 1 shows the number of remaining structures in each bin after these filterings. A few further PDB files had to be removed due to their incompatibility with DSSP. After these steps, each PDB-file was split into multiple models, and the accessible surface area was determined using DSSP for each model. For each residue in the protein chain, the average accessible surface area over all models was used. The final counts for each PDB-structure are shown in S1 Data. The format is explained in S1 Text.

### Calculation of contact based potential

To obtain estimates for the free energies of transferring specific amino acid types from the outside of the protein to the hydrophobic core, we used two approaches. The first approach is based on *contacts* between amino acids, and between amino acids and the solvent as in the work of Abeln and Frenkel [30]. This potential has been shown to give an appropriate distinction between the protein core and surface by simulation. The second approach uses the presence or absence of amino acids on the *surface* of the protein, providing a more direct way to obtain the hydrophobicty of each amino acids.

In the contact based approach, we calculate knowledge-based pair-potentials over the set of structures described above. The free energy estimates *ϵ*_*i*,*j*_ between amino acid types *i* and *j* can be calculated as:

$$\begin{array}{c} \epsilon_{i,j}=-kT\ln(\frac{c_{i,j}}{\omega_{i,j}}) \end{array}$$ (1)

where *c*_*i*,*j*_ are the number of contacts between amino acids type *i* and *j*, and where *ω*_*i*,*j*_ is the expected number of contacts. Note that here we are specifically interested in the case where one of the interaction partners is the solvent, i.e. *ϵ*_*i*,solvent_.

We can calculate the expected number of contacts, *ω*_*i*,*j*_, by considering the distribution of the amino acid types *i* and *j* in the set of protein structures:

$$\begin{array}{c} \omega_{i,j}=\frac{n_{i}q_{i}n_{j}q_{j}}{\sum_{k}q_{k}n_{k}} \end{array}$$ (2)

here *n*_*i*_ *q*_*i*_ is the total amount of contacts for type *i*, where *n*_*i*_ is the number of amino acid of type *i* and *q*_*i*_ is the coordination number, which we set to 4 for all amino acids to remain consistent with Abeln and Frenkel [30]. Note that the sum in denominator loops over all the amino acids and water (*k*). In practise the total number of contacts for an amino acid type *n*_*i*_ *q*_*i*_ can be calculated directly from the data.

The number of water contacts is estimated through the size of the surface accessible area for a residue as calculated by DSSP [47]. Note that for the water contact points, we do not consider real water molecules, but a surface area similar to the size of an amino acid. We estimate the number of contacts as the product between *q* = 4 and the fraction of exposed surface area *α*_*r*_ for residue *r*. Hence, based on the assumption that a residue can interact with four other residues, water contact points can be created. The fraction of exposed surface area, *α*_*r*_, is given by:

$$\begin{array}{c} \alpha_{r}=\frac{S_{r}}{\max\{S_{a(r)}\}} \end{array}$$ (3)

*S*_*r*_ is the solvent accessible area, calculated with the DSSP program, and *a*(*r*) is the amino acid type of residue *r*; $\max\{S_{a(r)}\}$ is the maximum accessible area in an unfolded chain for that amino acid type.

### Calculation of surface based potential

An alternative measure for hydrophobicity can be obtained by calculating the propensity for an amino acid to be on the surface. Classic amino acid propensities, which are for example used to describe the affinity for a certain secondary structure type, can be calculated through a simple ratio of fractions e.g. Chapter 12 of Ref. [48]. Here we use the structural classes buried and non-buried. To decide whether a residue (*r*) is buried, we use a cutoff: *α*_*r*_ < 7% [49]. We can calculate the propensity (*P*) for amino acids to be buried as:

$$\begin{array}{c} P_{a,b}=p_{a,b}/p_{b} \end{array}$$ (4)

where *P*_*a*,*b*_ stands for the propensity for an amino acid type, *a*, to be buried as indicated by the subscript *b*. Translating this into counts yields:

$$\begin{array}{c} p_{a,b}=\frac{N_{a,b}}{N_{a,b}+N_{a,nb}} \end{array}$$ (5)

where *N*_*a*,*b*_ is the total number of amino acids of type *a* that are buried, and *N*_*a*,*nb*_ is the total number of amino acids of type *a* that are non-buried. Similarly,

$$\begin{array}{c} p_{b}=\frac{N_{b}}{N_{b}+N_{nb}} \end{array}$$ (6)

where *N*_*b*_ is the total number of buried amino acids, and *N*_*nb*_ is the total number of amino acids that are not buried.

When propensities are used to estimate transfer free energies, through Δ*F*_*a*,*b*_ = −*kT* log(*P*_*a*,*b*_) it has the disadvantage that:

$$\begin{array}{c} \Delta F_{a,b}\neq -\Delta F_{a,nb} \end{array}$$ (7)

This can be seen by substituting the formula for *P*_*a*,*nb*_ in the formula for the free energy, Δ*F*.

Here we define our propensities in an alternative way to overcome this problem similar to Shatyan *et al*. [21]. If we define our alternative propensities, P*, analogous to a partition coefficient, we obtain:

$$\begin{array}{c} P_{a,b}^{*}=\frac{p_{a,b}^{*}}{p_{a,nb}^{*}}=\frac{N_{a,b}/N_{a,nb}}{N_{b}/N_{nb}} \end{array}$$ (8)

which does have the desired property summarized in Eq 7.

### Calculation of area based potential

While the contact based potential is established, some of the assumptions are particularly useful in the context of a coarse grained lattice simulation. On the other hand, the surface based potential uses the assumption that a residue is buried when less then 7% of its surface is exposed. To test the robustness of our results with regards to these assumptions, we investigated two additional potentials, based on the exposed *area*. The first one corresponds to the contact based potential, with very large (infinite) coordination numbers. This area based potential is calculated by comparing the amount of exposed surface area, *S*_*r*_, for an amino acid type *a* to that of the average amino acid.

$$C_{a}=-\log(\frac{N}{N_{a}}\frac{\sum_{i\in a}\frac{S_{r_{i},a}}{\max(S_{a(r_{i})})}}{\sum_{j}\frac{S_{r_{j}}}{\max(S_{r_{j}})}})$$ (9)

*S*_*r*_ is the solvent accessible area, calculated with the DSSP program, and *a*(*r*) is the amino acid type of residue *r*; $\max\{S_{a(r)}\}$ is the maximum accessible area in an unfolded chain for that amino acid type.

A similar potential, but scaled with the maximum solvent accessible area, is also calculated. We will refer to this potential as the scaled area based potential, *C*_*a*,*s*_ = *C*_*a*_ max(*S*_*a*_). The interactions of each residue are multiplied by its maximum accessible surface area. The results for this potential are very similar. Large residues have a higher interaction score when compared to smaller residues. The results for this potential are shown in S11, S12, S13 and S14 Figs.

### Significance of temperature dependence

The estimated error to the mean for each data point was obtained by splitting the data into five parts each containing an equal number of structures. The potential was recalculated for each of the five parts, and a standard deviation was calculated from each of them. This allows us to estimate a 95% confidence interval by taking two standard errors on each side of the mean. These are the error bars shown in the plots.

The significance of the temperature dependence of the potentials was determined through a resampling procedure for two different temperature bins: the lowest temperature range and room temperature. We resampled our data by shuffling the temperature labels of the protein structures and recalculating the contact based and surface based potentials for a set of 1000 random samples. P-values for the difference in hydrophobicity between the two temperature bins were determined as the fraction of resampled free energy differences that were larger in size than the original calculation.

### Fitting procedure

To obtain an estimate for the temperature dependence of the potential, we need to assign a single temperature for the structures within a temperature bin. The average temperature of the structures is taken to be the temperature of the bin. A weighted least squares fitting procedure was used to fit a parabola to the potential as a function of temperature, which is a reasonable approximation to the relation found in both theory and experiment. In a weighted least squares fit, the sum $S=\sum_{i=1}^{n}w_{i}r_{i}^{2}$ is minimized. Here, the *i* indicates the index of the temperature bin, *w*_*i*_ is the weight, and *r*_*i*_ is the difference between observations and the model. The number of residues of type *s* in bin *i* was used as weight.

## Supporting Information

S1 Data Data file containing counts. Counts of the different parameters, for each PDB-structure, in a tab-separated format.
(TXT)

S1 Text Description raw data, contained in S1 Data. (PDF)

S2 Text Order of magnitude estimation for temperature dependence of protein stability. (PDF)

S1 Fig Surface based free energy estimates for classes of amino acids reference corrected. Points show the free energy estimates for each temperature bin, lines are fitted with a parabola, consistent with the potentials found in [10].
(EPS)

S2 Fig Surface based free energy estimates for all amino acids reference corrected. Points show the free energy estimates for each temperature bin, lines are fitted with a parabola, consistent with the potentials found in [10].
(EPS)

S3 Fig Surface based free energy estimates for all amino acids not corrected. Points show the free energy estimates for each temperature bin, lines are fitted with a parabola, consistent with the potentials found in [10].
(EPS)

S4 Fig Contact based free energy estimates for classes of amino acids reference corrected. Points show the free energy estimates for each temperature bin, lines are fitted with a parabola, consistent with the potentials found in [10].
(EPS)

S5 Fig Contact based free energy estimates for all amino acids reference corrected. Points show the free energy estimates for each temperature bin, lines are fitted with a parabola, consistent with the potentials found in [10].
(EPS)

S6 Fig Contact based free energy estimates for all amino acids not corrected. Points show the free energy estimates for each temperature bin, lines are fitted with a parabola, consistent with the potentials found in [10].
(EPS)

S7 Fig Area based free energy estimates for classes of amino acids reference corrected. Points show the free energy estimates for each temperature bin, lines are fitted with a parabola, consistent with the potentials found in [10].
(EPS)

S8 Fig Area based free energy estimates for classes of amino acids not corrected. Points show the free energy estimates for each temperature bin, lines are fitted with a parabola, consistent with the potentials found in [10].
(EPS)

S9 Fig Area based free energy estimates for all amino acids reference corrected. Points show the free energy estimates for each temperature bin, lines are fitted with a parabola, consistent with the potentials found in [10].
(EPS)

S10 Fig Area based free energy estimates for all amino acids not corrected. Points show the free energy estimates for each temperature bin, lines are fitted with a parabola, consistent with the potentials found in [10].
(EPS)

S11 Fig Scaled area based free energy estimates for classes of amino acids reference corrected. Points show the free energy estimates for each temperature bin, lines are fitted with a parabola, consistent with the potentials found in [10].
(EPS)

S12 Fig Scaled area based free energy estimates for classes of amino acids not corrected. Points show the free energy estimates for each temperature bin, lines are fitted with a parabola, consistent with the potentials found in [10].
(EPS)

S13 Fig Scaled area based free energy estimates for all amino acids reference corrected. Points show the free energy estimates for each temperature bin, lines are fitted with a parabola, consistent with the potentials found in [10].
(EPS)

S14 Fig Scaled area based free energy estimates for all amino acids not corrected. Points show the free energy estimates for each temperature bin, lines are fitted with a parabola, consistent with the potentials found in [10].
(EPS)

S15 Fig Quadratic fits of the temperature dependence of LCW-theory for various sizes. The colored, dashed lines show theoretical predictions based on calculation from LCW-theory [10, 42]. The gray, solid lines, show a quadratic fit to these theoretical predictions.
(EPS)

## References

[1] Baldwin RL. Energetics of protein folding. J Mol Biol. 2007 8;371(2):283–301. Available from: http://www.sciencedirect.com/science/article/pii/S0022283607007371 http://www.ncbi.nlm.nih.gov/pubmed/17582437. 17582437 10.1016/j.jmb.2007.05.078

[2] Wood GR, Srivastava S, Patton Y, Fisher DW. Cotranslational protein folding and terminus hydrophobicity. Advances in Bioinformatics. 2011;2011 10.1155/2011/176813 21687643 PMC3112501

[3] Zarrine-Afsar A, Wallin S, Neculai AM, Neudecker P, Howell PL, Davidson AR, et al Theoretical and experimental demonstration of the importance of specific nonnative interactions in protein folding. Proceedings of the National Academy of Sciences. 2008;105(29):9999–10004. Available from: http://www.pnas.org/content/105/29/9999.abstract. 10.1073/pnas.0801874105 PMC2481363 18626019

[4] Widom B, Bhimalapuram P, Koga K. The hydrophobic effect. Phys Chem Chem Phys. 2003;5(15):3085 10.1039/b304038k

[5] Chandler D. Interfaces and the driving force of hydrophobic assembly. Nature. 2005 9;437(7059):640–647. Available from: 10.1038/nature04162. 10.1038/nature04162 16193038

[6] Rezus YLA, Bakker HJ. Observation of Immobilized Water Molecules around Hydrophobic Groups. Phys Rev Lett. 2007 10;99(14):148301 Available from: http://link.aps.org/doi/10.1103/PhysRevLett.99.148301. 10.1103/PhysRevLett.99.148301 17930728

[7] Hazy E, Bokor M, Kalmar L, Gelencser A, Kamasa P, Han KH, et al Distinct Hydration Properties of Wild-Type and Familial Point Mutant A53T of α-Synuclein Associated with Parkinson’s Disease. Biophysical Journal. 2011 9;101(9):2260–2266. Available from: http://www.cell.com/biophysj/abstract/S0006-3495%2811%2901061-7. 10.1016/j.bpj.2011.08.052 22067166 PMC3207174

[8] Rettich TR, Handa YP, Battino R, Wilhelm E. Solubility of gases in liquids. 13. High-precision determination of Henry’s constants for methane and ethane in liquid water at 275 to 328 K. J Phys Chem. 1981;85(22):3230–3237. Available from: http://pubs.acs.org/doi/abs/10.1021/j150622a006. 10.1021/j150622a006

[9] Kim A, Szoka FC. Amino acid side-chain contributions to free energy of transfer of tripeptides from water to octanol. Pharm Res. 1992 4;9(4):504–14. Available from: http://www.ncbi.nlm.nih.gov/pubmed/1495896. 10.1023/A:1015892313856 1495896

[10] Huang DM, Chandler D. Temperature and length scale dependence of hydrophobic effects and their possible implications for protein folding. Proc Natl Acad Sci. 2000;97(15):8324–8327. Available from: http://www.pnas.org/content/97/15/8324.abstract. 10.1073/pnas.120176397 10890881 PMC26946

[11] Vajpai N, Nisius L, Wiktor M, Grzesiek S. High-pressure NMR reveals close similarity between cold and alcohol protein denaturation in ubiquitin. Proceedings of the National Academy of Sciences. 2013;110(5):E368–E376. Available from: http://www.pnas.org/content/110/5/E368.abstract. 10.1073/pnas.1212222110 PMC3562818 23284170

[12] Dias CL, Ala-Nissila T, Karttunen M, Vattulainen I, Grant M. Microscopic Mechanism for Cold Denaturation. Phys Rev Lett. 2008 3;100(11):1–4. Available from: http://link.aps.org/doi/10.1103/PhysRevLett.100.118101. 10.1103/PhysRevLett.100.118101 18517830

[13] Uversky VN, Li J, Fink AL. Evidence for a Partially Folded Intermediate in α-Synuclein Fibril Formation. Journal of Biological Chemistry. 2001;276(14):10737–10744. Available from: http://www.jbc.org/content/276/14/10737.abstract. 10.1074/jbc.M010907200 11152691

[14] Frock A, Kelly R. Extreme thermophiles: moving beyond single-enzyme biocatalysis. Curr Opin Chem Eng. 2012;1(4):363–372. Available from: http://www.sciencedirect.com/science/article/pii/S2211339812000433. 10.1016/j.coche.2012.07.003 23413412 PMC3568776

[15] Tajima T, Fuki K, Kataoka N, Kudou D, Nakashimada Y, Kato J. Construction of a simple biocatalyst using psychrophilic bacterial cells and its application for efficient 3-hydroxypropionaldehyde production from glycerol. AMB Express. 2013 1;3(1):69 Available from: http://www.ncbi.nlm.nih.gov/pubmed/24314120. 10.1186/2191-0855-3-69 24314120 PMC4029479

[16] Folch B, Dehouck Y, Rooman M. Thermo- and mesostabilizing protein interactions identified by temperature-dependent statistical potentials. Biophys J. 2010 2;98(4):667–77. Available from: http://www.pubmedcentral.nih.gov/articlerender.fcgi?artid=2820637&tool=pmcentrez&rendertype=abstract. 10.1016/j.bpj.2009.10.050 20159163 PMC2820637

[17] Pucci F, Rooman M. Stability Curve Prediction of Homologous Proteins Using Temperature-Dependent Statistical Potentials. PLoS Comput Biol. 2014;10(7):e1003689 Available from: http://dx.doi.org/10.1371%2Fjournal.pcbi.1003689. 10.1371/journal.pcbi.1003689 25032839 PMC4102405

[18] Kyte J, Doolittle RF. A simple method for displaying the hydropathic character of a protein. J Mol Biol. 1982 5;157(1):105–32. Available from: http://www.ncbi.nlm.nih.gov/pubmed/7108955. 10.1016/0022-2836(82)90515-0 7108955

[19] Chothia C. The nature of the accessible and buried surfaces in proteins. J Mol Biol. 1976 7;105(1):1–12. Available from: http://www.ncbi.nlm.nih.gov/pubmed/994183. 10.1016/0022-2836(76)90191-1 994183

[20] Rose GD, Geselowitz AR, Lesser GJ, Lee RH, Zehfus MH. Hydrophobicity of amino acid residues in globular proteins. Science (80-). 1985;229(4716):834–838. Available from: http://www.sciencemag.org/content/229/4716/834.abstract. 10.1126/science.4023714 4023714

[21] Shaytan AK, Shaitan KV, Khokhlov AR. Solvent accessible surface area of amino acid residues in globular proteins: correlation of apparent transfer free energies with experimental hydrophobicity scales. Biomacromolecules. 2009 5;10(5):1224–37. Available from: http://www.ncbi.nlm.nih.gov/pubmed/19334678. 10.1021/bm8015169 19334678

[22] Venselaar H, Te Beek TaH, Kuipers RKP, Hekkelman ML, Vriend G. Protein structure analysis of mutations causing inheritable diseases. An e-Science approach with life scientist friendly interfaces. BMC Bioinformatics. 2010 1;11(1):548 Available from: http://www.pubmedcentral.nih.gov/articlerender.fcgi?artid=2992548&tool=pmcentrez&rendertype=abstract. 10.1186/1471-2105-11-548 21059217 PMC2992548

[23] Floris M, Raimondo D, Leoni G, Orsini M, Marcatili P, Tramontano A. MAISTAS: a tool for automatic structural evaluation of alternative splicing products. Bioinformatics. 2011 6;27(12):1625–9. Available from: http://www.pubmedcentral.nih.gov/articlerender.fcgi?artid=3106191&tool=pmcentrez&rendertype=abstract. 10.1093/bioinformatics/btr198 21498402 PMC3106191

[24] Oldfield CJ, Cheng Y, Cortese MS, Brown CJ, Uversky VN, Dunker AK. Comparing and combining predictors of mostly disordered proteins. Biochemistry. 2005 2;44(6):1989–2000. Available from: http://www.hubmed.org/display.cgi?uids=15697224. 10.1021/bi047993o 15697224

[25] Cilia E, Pancsa R, Tompa P, Lenaerts T, Vranken WF. From protein sequence to dynamics and disorder with DynaMine. Nat Commun. 2013 11;4 Available from: 10.1038/ncomms374110.1038/ncomms3741. 10.1038/ncomms374110.1038/ncomms3741 24225580

[26] Zhou H, Zhou Y. Distance-scaled, finite ideal-gas reference state improves structure-derived potentials of mean force for structure selection and stability prediction. Protein Sci. 2002;11(11):2714–2726. Available from: 10.1110/ps.0217002. 10.1110/ps.0217002 12381853 PMC2373736

[27] Buchete NV, Straub JE, Thirumalai D. Development of novel statistical potentials for protein fold recognition. Curr Opin Struct Biol. 2004;14(2):225–232. Available from: http://www.sciencedirect.com/science/article/pii/S0959440X04000351. 10.1016/j.sbi.2004.03.002 15093838

[28] Miyazawa S, Jernigan RL. Estimation of effective interresidue contact energies from protein crystal structures: quasi-chemical approximation. Macromolecules. 1985;18:534–552. 10.1021/ma00145a039

[29] Betancourt MR, Thirumalai D. Pair potentials for protein folding: choice of reference states and sensitivity of predicted native states to variations in the interaction schemes. Protein Sci. 1999 2;8(2):361–369. Available from: http://www.hubmed.org/display.cgi?uids=10048329 http://www.pubmedcentral.nih.gov/articlerender.fcgi?artid=2144252&tool=pmcentrez&rendertype=abstract. 10.1110/ps.8.2.361 10048329 PMC2144252

[30] Abeln S, Frenkel D. Accounting for protein-solvent contacts facilitates design of nonaggregating lattice proteins. Biophys J. 2011 2;100(3):693–700. Available from: http://www.pubmedcentral.nih.gov/articlerender.fcgi?artid=3030183&tool=pmcentrez&rendertype=abstract. 10.1016/j.bpj.2010.11.088 21281584 PMC3030183

[31] Shen My, Sali A. Statistical potential for assessment and prediction of protein structures. Protein Sci. 2006;15:2507–2524. Available from: http://onlinelibrary.wiley.com/doi/10.1110/ps.062416606/full. 10.1110/ps.062416606 17075131 PMC2242414

[32] Coluzza I, Muller HG, Frenkel D. Designing refoldable model molecules. Phys Rev E. 2003 10;68(4):46703 10.1103/PhysRevE.68.046703 14683075

[33] Coluzza I, Frenkel D. Monte Carlo Study of Substrate-Induced Folding and Refolding of Lattice Proteins. Biophysical Journal. 2007;92(4):1150–1156. Available from: http://www.sciencedirect.com/science/article/pii/S0006349507709268. 10.1529/biophysj.106.084236 17142295 PMC1783883

[34] Ni R, Abeln S, Schor M, Cohen Stuart MA, Bolhuis PG. Interplay between Folding and Assembly of Fibril-Forming Polypeptides. Phys Rev Lett. 2013 7;111(5):058101 Available from: http://link.aps.org/doi/10.1103/PhysRevLett.111.058101. 10.1103/PhysRevLett.111.058101 23952447

[35] Abeln S, Vendruscolo M, Dobson CM, Frenkel D. A Simple Lattice Model That Captures Protein Folding, Aggregation and Amyloid Formation. PLoS One. 2014 1;9(1):e85185 Available from: http://dx.plos.org/10.1371/journal.pone.0085185. 10.1371/journal.pone.0085185 24454816 PMC3893179

[36] Halperin I, Ma B, Wolfson H, Nussinov R. Principles of docking: An overview of search algorithms and a guide to scoring functions. Proteins. 2002 6;47(4):409–43. Available from: http://www.ncbi.nlm.nih.gov/pubmed/12001221. 10.1002/prot.10115 12001221

[37] Berman HM, Westbrook J, Feng Z, Gilliland G, Bhat TN, Weissig H, et al The Protein Data Bank. Nucleic Acids Res. 2000 1;28(1):235–242. 10.1093/nar/28.1.235 10592235 PMC102472

[38] Thiriot DS, Nevzorov AA, Opella SJ. Structural basis of the temperature transition of Pf1 bacteriophage. Protein Sci. 2005;14(4):1064–1070. Available from: 10.1110/ps.041220305. 10.1110/ps.041220305 15741342 PMC2253442

[39] Shirota M, Ishida T, Kinoshita K. Analyses on hydrophobicity and attractiveness of all-atom distance-dependent potentials. Protein Sci. 2009;18(9):1906–1915. Available from: 10.1002/pro.201. 10.1002/pro.201 19588493 PMC2777365

[40] Wuttke R, Hofmann H, Nettels D, Borgia MB, Mittal J, Best RB, et al Temperature-dependent solvation modulates the dimensions of disordered proteins. Proceedings of the National Academy of Sciences. 2014;111(14):5213–5218. Available from: http://www.pnas.org/content/111/14/5213.abstract. 10.1073/pnas.1313006111 PMC3986154 24706910

[41] Chamberlin AC, Cramer CJ, Truhlar DG. Predicting Aqueous Free Energies of Solvation as Functions of Temperature. The Journal of Physical Chemistry B. 2006;110(11):5665–5675. Available from: 10.1021/jp057264y. 10.1021/jp057264y 16539512

[42] Lum K, Chandler D, Weeks JD. Hydrophobicity at Small and Large Length Scales. The Journal of Physical Chemistry B. 1999;103(22):4570–4577. Available from: http://pubs.acs.org/doi/abs/10.1021/jp984327m. 10.1021/jp984327m

[43] Gast K, Damaschun H, Misselwitz R, Müller-Frohne M, Zirwer D, Damaschun G. Compactness of protein molten globules: temperature-induced structural changes of the apomyoglobin folding intermediate. European Biophysics Journal. 1994;23(4):297–305. Available from: 10.1007/BF00213579. 10.1007/BF00213579 7805629

[44] Hobohm U, Scharf M, Schneider R, Sander C. Selection of representative protein data sets. Protein Sci. 1992;1(3):409–417. 10.1002/pro.5560010313 1304348 PMC2142204

[45] Hobohm U, Sander C. Enlarged representative set of protein structures. Protein Sci. 1994 3;3(3):522–524. Available from: http://www.hubmed.org/display.cgi?uids=8019422. 10.1002/pro.5560030317 8019422 PMC2142698

[46] Griep S, Hobohm U. PDBselect 1992–2009 and PDBfilter-select. Nucleic Acids Res. 2010;38(suppl 1):D318–D319. 10.1093/nar/gkp786 19783827 PMC2808879

[47] Kabsch W, Sander C. Dictionary of protein secondary structure: pattern recognition of hydrogen-bonded and geometrical features. Biopolymers. 1983;22(12):2577–2637. 10.1002/bip.360221211 6667333

[48] Zvelebil MJ, Baum JO. Understanding Bioinformatics. Garland Science; 2008 Available from: http://books.google.nl/books?id=dGayL_tdnBMC.

[49] Hubbard TJ, Blundell TL. Comparison of solvent-inaccessible cores of homologous proteins: definitions useful for protein modelling. Protein Eng. 1987 6;1(3):159–171. Available from: http://www.hubmed.org/display.cgi?uids=3507702. 10.1093/protein/1.3.159 3507702

