# Supplementary material for: The Hydrophobic Temperature Dependence of Amino Acids Directly Calculated from Protein Structures
Source: PLoS Comput Biol. 2015 May 22;11(5):e1004277. doi: 10.1371/journal.pcbi.1004277 (PMC4441443; doi:10.1371/journal.pcbi.1004277)
Supplement: S1 Text — (PDF) [file pcbi.1004277.s002.pdf]

S1 Text: Description statistics file

Raw counts for each PDB structure per amino acid can be found at `http://www.few.vu.nl/~abeln/hydrophobicT/`. Each row corresponds to a PDB structure. Each column corresponds to a feature of the PDB structure. The identifiers in de header are described below. The statistics are provided per amino acid type, which is indicated by its three letter-code. For example, Alanine is abbreviated as ALA.

- PDB_chainID: 4-Letter pdb code followed by underscore and one letter indicating which chain is used
- Temperature: Temperature in Kelvin
- XXX_bur: Number of buried residues of type XXX. Buried is defined as less than 7% solvent accessible area.
- XXX_unbur: Number of exposed residues of type XXX. Exposed is defined as more than 7% solvent accessible area.
- XXX_Rsas: The relative solvent accesible area of type XXX. Is defined as accessible_ area/max_solvent accessible area
- XXX_Choh: Number of contacts with water for a residues of type XXX. Is defined as round($4 \times$ accessible area/max_solvent_accessible_area)
- XXX_Caa: Number of contacts with other residues for a residues of type XXX. Is defined as 4 - XXX_Choh.
