# Supplementary material for: The Hydrophobic Temperature Dependence of Amino Acids Directly Calculated from Protein Structures
Source: PLoS Comput Biol. 2015 May 22;11(5):e1004277. doi: 10.1371/journal.pcbi.1004277 (PMC4441443; doi:10.1371/journal.pcbi.1004277)
Supplement: S2 Text — (PDF) [file pcbi.1004277.s003.pdf]

S2 Text: Order of magnitude estimation for temperature dependence of protein stability

To determine whether our potential yields results that are of the right order of magnitude, we will consider energy contributions for a typically sized protein. A typical protein domain has around 200 residues containing roughly 60 hydrophobic residues that are buried upon folding. Stabilities range typically between 5-20 kT at room temperature. Our potentials show that, for a hydrophobic residue the effective interaction decreases by about 0.1-0.4 kT between room temperature and 5 °C. This will yield a stability between 14 kT and -19 kT for such a protein at 5 °C. We therefore estimate that some proteins may start cold denaturating just above the freezing point of water. Hence the decrease in hydrophobic effect of 0.1-0.4 kT for this temperature range appears to be consistent with cold denaturation behaviour of real proteins: most proteins sporadically cold denature above the freezing point of water [1].

Many factors not considered in this calculation will also influence the folding. For example, the internal contacts have a significant temperature dependence [2]. Moreover, the chain entropy is less important at low temperatures, compensating partially for the lost stability of the native state. These and other factors are different for each protein and difficult to estimate.

References

1. Vajpai N, Nisius L, Wiktor M, Grzesiek S (2013) High-pressure NMR reveals close similarity between cold and alcohol protein denaturation in ubiquitin. Proceedings of the National Academy of Sciences 110: E368–E376.

2. Pucci F, Rooman M (2014) Stability Curve Prediction of Homologous Proteins Using Temperature-Dependent Statistical Potentials. PLoS Comput Biol 10: e1003689.
